# Treating war- and conflict-related nightmares in children and youth: outcomes of a school-based intervention

**DOI:** 10.3389/frsle.2025.1658555

**Published:** 2026-01-08

**Authors:** Jon Håkon Schultz, June Forsberg, Eva Alisic, Safwat Diab, Gerlinde Harb

**Affiliations:** 1 UiT The Arctic University of Norway, Tromsø, Norway; 2 The University of Melbourne School of Languages and Linguistics, Parkville, VIC, Australia; 3 Al-Quds University, Abu Dis, Palestine

**Keywords:** posttraumatic nightmares, PTSD, sleep, stress, treatment

## Abstract

**Introduction:**

Recurrent nightmares often severely impair the quality of life, school functioning, and daily functioning of trauma-exposed children and adolescents. However, research to date is limited for treatments focused on reducing the impact of posttraumatic nightmares among youth in conflict zones. This study aimed to investigate the outcome of the Better Learning Program 3 (BLP) as it was implemented in over 100 schools in Gaza (2012–2017).

**Methods:**

Treatment outcome was investigated in an open trial among war- and conflict-exposed students (6–17 years of age) in Gaza (*N* = 1093). All participants sought help with nightmares and sleep disturbance and reported recurrent traumatic nightmares on average 4.86 nights per week. The intervention was inspired by imagery rehearsal therapy and trauma-focused cognitive behavioral therapy.

**Results:**

Students experienced significant week-to-week reductions in reported nightmares throughout the 8-week intervention. Approximately half of the participants reported no nightmares post-treatment, and a further 47% reported a reduction in nightmare frequency. In a 10-month follow-up, 42% of a smaller sample (*n* = 215) maintained treatment gains and remained free of nightmares, whereas 42% maintained a reduction of nightmares to one or two per week. Students with relapse responded effectively to booster sessions offered after follow-up.

**Discussion:**

The current study demonstrates the apparent success of this school-based treatment, showing that it is both feasible to intervene directly with students' nightmare disturbance and to significantly reduce their nightmare frequency.

## Introduction

Posttraumatic nightmares are often referred to as a hallmark of posttraumatic stress disorder (PTSD; e.g., Ross et al., 1989; El-Solh, 2018) and a symptom that is both impairing by itself as well as closely related to other posttraumatic symptomatology. Nightmares are distressing dreams that wake the dreamer, and they often replay traumatic events or incorporate feelings experienced during the trauma, such as helplessness and horror. Nightmares cause sleep disruption and sleep loss, resulting in daytime impairment in functioning as well as increased reexperiencing symptoms during the daytime. In addition, frequent posttraumatic nightmares are commonly associated with high PTSD severity, depression, and suicidality (Levin and Nielsen, 2007; Bernert et al., 2005).

Although the vast majority of research on sleep and nightmares has studied adults, a review of studies in children concluded that exposure to traumatic events is linked to sleep disturbance in children (Kovachy et al., 2013), highlighting the importance of studies of pediatric trauma-related nightmares. While some idiopathic nightmares in children can be developmentally appropriate and vary with age (Schredl and Reinhard, 2011; El Sabbagh et al., 2023), nightmares after a traumatic event can more severely disrupt sleep and indicate other posttraumatic difficulties. In fact, children and adolescents with a trauma history report much more frequent nightmares (9.7 per month) than non-traumatized controls (1.7 per month; Ossa et al., 2013). Irrespective of whether children are given a PTSD diagnosis, posttraumatic sleep symptoms such as nightmares are frequent in trauma-exposed children (Wamser-Nanney and Chesher, 2017) and trauma-exposed migrant youth (Bryant, 2024). Children with such highly distressing posttraumatic nightmares exhibit distress upon waking, sleep loss, and impairment in daytime and school functioning (Schultz et al., 2021). Moreover, studies have demonstrated that the frequency of nightmares in children and youth is strongly related to other impairing posttraumatic symptoms (Secrist et al., 2019). Research has furthermore demonstrated a relationship between the severity of traumatization and the probability of posttraumatic nightmares (Nader, 1996), and between the number of trauma types experienced by youth and the likelihood of impairing nightmares (Secrist et al., 2019). Multiple trauma exposures or experiences of chronic traumatization may have a cumulative impact on children's symptoms, their severity, and resulting long-term impairment.

Armed conflict has many adverse effects on exposed children, even more so if the conflict is protracted and traumatic experiences recur. Children experience both direct and indirect effects, and the harm can be seen acutely as well as long-term (Kadir et al., 2019). This can be witnessed in the mental health of children living in Gaza over the last two decades. The military siege of Gaza has been enforced from 2007 until the currently 2023–25 war, with control of the movement of goods and people causing deprivation of basic needs. Since the start of the siege, the people of Gaza have experienced five major wars and scaled-up conflicts: in 2008–2009, 2012, 2014, 2016, and 2021 (United Nations Human Rights Council (UNHRC), 2015, 2022). The current war followed the Hamas attack against Israel on October 7, 2023. Prior to this war, several studies have documented that young people in Gaza had experienced dramatically high exposure to potentially traumatic events and stressful conditions that could trigger sleep disturbance and nightmares. In fact, estimates of PTSD prevalence range from 23 to 70% in Palestinian children (Dimitry, 2011), and a recent nightmare prevalence study in Gaza documented that 56% of 10–12-year-old students reported recurrent nightmares, with an average of 4.2 nights with nightmares and a mean duration of approximately 2.5 years (Schultz et al., 2021). The distressing nature of such nightmares was highlighted in a study of 64 youths in Gaza, ages 12–16, who had lived through three wars and experienced ongoing conflict and political insecurity (Harb and Schultz, 2020). Students reported on average five nightmares per week for an average of 3 years, and they reported disrupted sleep, fear of going to sleep, not feeling rested in the morning, as well as impaired academic functioning and daytime sleepiness. Furthermore, the content of their nightmares demonstrated frightening themes of being under attack and loss of self-efficacy/control; threat levels were high, and almost 60% included the threat of death in the nightmares (Harb and Schultz, 2020). A larger study (*N* = 1,093; Schultz et al., 2021) found an association between nightmares and reduced self-reported academic functioning: students reporting high nightmare intensity and a longer duration experienced a more severe reduction in their academic functioning. The nightmare disturbances created substantial challenges for these students in the learning process and their chances for successfully completing their education. Further, students experiencing ongoing conflict emerge as particularly vulnerable to recurrent posttraumatic nightmares due to the combination of the continuing occurrence of potentially traumatic events and ongoing sleep deprivation affecting their academic and psychiatric functioning (Schultz et al., 2021). The high incidence of recurrent posttraumatic nightmares documented in the conflict-affected youth population in Gaza poses severe potential threats to their mental health, development, as well as daily functioning and school functioning. Very few studies examined the treatment of traumatic nightmares in children and adolescents (Fernandez et al., 2013; Krakow et al., 2001), however, recent years have shown increased research attention in this area such as a case study of cognitive behavioral treatment for idiopathic nightmares (DeMarni Cromer et al., 2022) and an RCT of a telehealth treatment for children (Cromer et al., 2024). Research in conflict-affected populations is lacking, and even less is known regarding conflict-affected populations still residing within conflict areas. It is likely, as has been suggested in adults (Harb et al., 2009), that focusing on the treatment of posttraumatic sleep problems may prove to be a relatively easy entry into the treatment of posttraumatic stress symptoms and may have added effects on ameliorating other posttraumatic symptomatology. Behavioral interventions directly addressing nightmares in traumatized children have great potential as a first-line treatment for a core problem in posttraumatic adjustment, as many studies in adults have shown them to be effective in reducing nightmare frequency and distress (e.g., Augedal et al., 2013; Gill et al., 2023; Casement and Swanson, 2012). Additionally, sleep- and nightmare-focused interventions allow for straightforward identification of a clinical population in schools due to easily assessed symptoms. Sleep symptoms additionally carry less cultural stigma for students and their families, which is often common with regards to mental health treatment (e.g., Bruno et al., 2019).

UiT, the Arctic University of Norway, together with the Norwegian Refugee Council (NRC), developed a traumatic-nightmare-focused, school-based intervention, the Better Learning Program 3 (Norwegian Refugee Council, 2011: 2nd ed.), in response to the needs of traumatized youths in Gaza and the West Bank. The Better Learning Program (BLP) utilizes strategies of psychoeducation about trauma and traumatic reactions, relaxation training, nightmare exposure, and nightmare rescripting and has been implemented in schools in Gaza since 2012. The implementation was carried out by NRC in close collaboration with the United Nations Relief and Works Agency for Palestine Refugees in the Near East (UNRWA). The current report examines the program's treatment outcomes.

Specifically, the current study aimed to document and explore students' treatment outcomes as evidenced by changes in traumatic nightmare frequency and intensity, as well as general wellbeing post-treatment. The research questions were formulated as follows:

- Does nightmare frequency decrease from pre- to post-treatment?
- How does nightmare frequency change over the course of the treatment?
- Is the change in nightmare frequency and intensity related to gender, age, or the baseline severity of nightmare symptoms?
- Does general wellbeing increase after treatment?
- What characteristics describe the non-responders?

## Materials and methods

### Participants

A total of 1,093 students (594 girls and 504 boys; age range 6–17: 179 between 6 and 9, 673 between 10 and 12, and 241 between 13 and 17 years old; mean age 11.07: SD = 1.77) participated in five different cohort studies between 2012 and 2017. These cohorts together are described here as one open trial. During this period, all students found eligible for the intervention were included in the data collection. Participants were recruited from 108 schools geographically spread across the entire area of Gaza. Due to an administrative error during baseline registry, 13 respondents' data were missing nightmare frequency, leaving 1,080 (96.5%) respondents with a recorded number of nights with nightmares. A demographic overview of all participants in each cohort is presented in Table 1.

**Table 1:** Demographic characteristics of the participants by cohort (*N* = 1,093).

**Cohort**	***N***	**Females %**	**Mean age (SD)**	***N* Age 6–9**	***N* Age 10–12**	***N* Age 13–17**
Cohort 1 (2012)	63	24/38.1%	10.57 (2.01)	30	22	11
Cohort 2 (2013)	160	122/76.3%	11.19 (1.52)	27	108	25
Cohort 3 (2014)	111	31/27.9%	10.95 (2.05)	12	78	21
Cohort 4 (2015)	364	179/49.2%	11.61 (1.83)	49	201	114
Cohort 5 (2016)	395	238/60.5%	11.02 (1.60)	61	264	70
Total	1093	594/54.4%	11.07 (1.77)	179	673	241
Cohort 5 follow-up (2017)	215	145/67.4%	11.20 (1.60)	23	153	39

### Procedure

The BLP is a school-based mental health and psychosocial program (MHPSS) consisting of three interventions that can be used separately or together as a stepped care approach. BLP-1 is the first-level intervention and applies a universal approach targeting all students affected by crisis (Norwegian Refugee Council, 2025: 5th ed.). The second intervention, BLP-2, targets students with impaired school functioning due to high levels of stress (Norwegian Refugee Council, 2022, 3rd ed.), while BLP-3 (Norwegian Refugee Council, 2011: 2nd ed.) is an indicated intervention for students with persistent posttraumatic nightmares. This study reports on the implementation of BLP-3 as a stand-alone school-based intervention starting in 2011 in Gaza. Inclusion criteria for the BLP-3 program were as follows: (a) having nightmares related to a traumatic event, with two or more nights per week; (b) the nightmares had persisted for 3 months or more; and (c) nightmares impaired school or daily functioning.

Bachelor's-level counselors at each school were trained in the recruitment and screening procedures and led the screening interviews. The counselors gave briefings in classrooms, including brief psychoeducation about posttraumatic nightmare experiences (e.g., stating that nightmares are normal reactions that can be dealt with), and invited students with recurrent trauma-related nightmares to sign up for the program to reduce their nightmares. Interested students were screened either individually or in small groups using a structured interview assessing nightmare frequency, intensity, duration, and content as well as general wellbeing. To ensure a homogeneous sample of conflict-related nightmares, respondents who mentioned domestic violence or sexual abuse as the sole source of their nightmares were given individual treatment and not included in treatment groups or in this study. Students' treatment attendance, dropout, and possible additional sessions were recorded by each counselor. The protocol allowed for up to two additional individual sessions if needed. The treatment group size ranged from 5 to 10, with a median group size of 8, and dropout during treatment was 5%.

Two cohorts included additional assessments and/or treatment. First, about 7 weeks after the end of treatment of Cohort 3, the 50-day armed conflict broke out in 2014. Due to this war's effect on the Gaza population, counselors reached out to students (*n* = 46) to provide four group-based booster sessions of 30–45 min at 4, 8, 12, and 23 weeks after the conflict and assessed students' nightmare frequency. The counselors were instructed to run sessions reinforcing the use of relaxation exercises, group sharing of nightmare frequency and intensity, sharing effective methods for stress reduction, and building self-efficacy and future hope. Second, Cohort 5 included a follow-up assessment: counselors contacted respondents 10 months post-treatment to assess their nightmare frequency and provide advice if nightmares had returned. Follow-up data were obtained from 215 of 395 students initially treated in Cohort 5. In most cases, students' loss to follow-up was due to their change of schools following the summer break, which made them hard to reach.

### The BLP-3 treatment protocol

The BLP design is theoretically based on the five principles for trauma recovery (Hobfoll et al., 2007) and inspired by three evidence-based treatment programs: trauma-focused cognitive behavioral therapy (TF-CBT: Cohen et al., 2012), narrative exposure therapy (NET: Schauer et al., 2005), and imagery rehearsal therapy (IRT; Harb et al., 2009; Pruiksma et al., 2025). BLP-3 has a clearly defined treatment protocol describing four group and four individual sessions consisting of (1) psychoeducation and normalization of stress reactions, (2) relaxation techniques, (3) coping skills enhancement, (4) social support, (5) parent involvement, (6) exposure by retelling and drawing the content of the traumatic nightmare, and (7) imagery rescripting and rehearsal (Norwegian Refugee Council, 2011, 2025). While developed in Northern Uganda, the intervention was contextualized and piloted in Gaza in close collaboration with UNRWA resulting in a revision of the manual for use in Gaza (2011).

The protocol is presented as a manual with a section describing the treatment rationale and a method section describing the session-by-session details of how to use the methods. Each of the eight sessions is manualized with a detailed structure, with a timetable indicating the time spent on each part of the session. Bachelor's-level school counselors led the treatment and were assisted by teachers, who also received basic training for the intervention. During the first two cohorts only, treatment was occasionally led by two teachers.

During the four group sessions, the participating children learn about and discuss their stress reactions, and they identify and draw a picture of their “worst” nightmare. In the individual sessions, they construct a timeline of their life, indicating and discussing the good and bad events of their lives with the counselor. The traumatic event(s) underlying the traumatic nightmare are identified, the content of the nightmare is discussed, and the story of the nightmare is changed with techniques of imagery rehearsal, producing a new dream drawing.

Psychological safety protocols during exposure and rescripting were an important part of formal training and supervision. Counselors were instructed in several main principles in this safety protocol: first, students should never be pressured, only encouraged during session activities; second, students with strong emotional reactions during or after the sessions should be followed up outside of sessions; third, counselors should seek advice when unclear about students' reactions; fourth, counselors should demonstrate awareness of when to refer students to a higher level of care and of possible referral pathways. Counselors furthermore received a 5-day basic training course, including 3 days of lectures and role play followed by 2 days of case presentations after gaining practical experience in BLP-3. All counselors were supervised by a line manager and had access to a BLP-3 instructor for discussing case management. Measures to prevent the potential secondary traumatization of counselors were provided by UNRWA through their support structure of their employees.

### Fidelity

Fidelity was assessed through formal and informal interviews with treatment providers by BLP-3 staff. In each cohort, samples of counselors were interviewed for fidelity checks. These interviews were conducted by representatives from UNRWA, NRC educational officers, and the first and fourth authors. Fidelity checks varied in terms of frequency, scope and exact questions used; however, all checks used a condensed quantitative fidelity checklist made for this study to assess protocol fidelity including assessments of exposure/rescripting elements and students' participation. Fidelity checks showed that the session structure of the BLP-3 treatment was followed, and the treatment protocol was reported to be easy to follow and was adhered to as a treatment manual. However, at the beginning of the 4-year intervention period, a tendency to conduct less strict exposure than initially assigned in the protocol was observed in fidelity checks. A somewhat less systematic *in-vivo* exposure to traumatic memories underlying the nightmare was conducted, or exposure was omitted from the treatment altogether. Counselors had either made a clinical judgment that the students were not ready for or did not need exposure, or the counselors were unsure of how and when to use exposure. The treatment protocol was subsequently slightly adjusted (in 2013), emphasizing that it allows for the use of gradual stepped exposure already built into the treatment rather than *in-vivo* exposure, placing more focus on elements of IRT through guided imagery for changing the nightmare drawing.

In-depth interviews with the six counselors who conducted booster sessions in Cohort 3 revealed that the counselors followed the session structure for group booster sessions. However, due to logistical challenges, 9 out of 46 students had to receive individual sessions instead of group sessions. During booster sessions, all children were eager to attend when invited and expressed relief and willingness to share their use of the various strategies, their symptoms, and their observed change in symptoms over the weeks.

### Ethical considerations

Participation in the nightmare intervention was voluntary. However, students were informed that they needed their parents' consent for participation, and if they wanted to withdraw from the sessions, they would need to discuss this with the counselor. Enrollment in the intervention and participation in the research required written parental consent after participating in an information meeting hosted by the school. Students gave their informed consent after receiving a detailed explanation of the study's aims, anonymization, and the possibility of withdrawing. Parents and/or students could opt out of participating in the research and still receive the treatment. UNRWA approved the treatment to be run as a pilot program in their school-based mental health service and reviewed and approved the ethics and procedures of the study protocol. Data were collected by UNRWA and NRC; names initially kept by UNRWA were deleted when unique ID numbers were assigned. The process of transferring data to UiT was approved by its ethics committee. This study was conducted in accordance with the Declaration of Helsinki.

### Measures

The self-report measures administered for this research were developed for this project and were kept brief due to the time limitations related to the school-based intervention. All questionnaires were translated from English to Arabic and then back-translated by certified translators.

The presence of nightmares and their frequency were measured with two items: “I have nightmares” (Yes/No) and “In the last week, how many nights with nightmares did you have in total?” The questionnaire measured current nightmares as opposed to lifetime prevalence of “ever having had a nightmare.” Nightmare duration was assessed on a 7-point scale: “How long have you experienced nightmares?” (0 = less than a year, 1 = 1 year … to 6 = more than 5 years). In Cohorts 1–4, nightmare intensity was measured with two dichotomous (yes/no) items: “Do you see pictures of the nightmare during the day?” and “Can you usually go to sleep after the nightmare?” In Cohort 5, nightmare intensity was also measured with three items adapted from the Nightmare Intensity Scale (Harb et al., manuscript in preparation) to assess the intensity of imagery, sensory details, and emotional experience in nightmares: “Is the nightmare vivid in terms that the picture is real and comes with details?,” “Are the bodily sensations in the nightmare vivid in terms that you can feel details in smell, voices, or noise,?” and “I have strong feelings during the nightmare.” The items were rated on a 6-point scale (0 = never, 1 = sort of, 2 = a little, 3 = moderately, 4 = a lot, 5 = very much), with a total score ranging from 0 to 5.

All students indicated whether their nightmares were linked to their worst experience, whether the nightmares reflected actual events that had happened to them, and whether the same nightmares recurred again and again. Trauma exposure was not formally assessed in the questionnaire. However, it was assessed clinically, first during the screening interview and then during treatment when pupils were constructing their timeline.

Help-seeking activity was measured with one item: “Have you told your teacher about your nightmares”? (yes/no). In Cohorts 3–5, general wellbeing was also measured with one item: “I am satisfied with my life most of the time” (yes/no).

### Statistical analysis

All statistical analyses were performed with SPSS 28.0 (IBM, SPSS, Chicago, IL). Nightmare duration, frequency, and intensity (only in Cohort 5) were assessed with descriptive statistics. A chi-square test was used to evaluate differences in gender, and differences between age groups were tested with analysis of variance (ANOVA). Histograms of residuals and Q-Q plots were examined to explore normality and normal distribution at baseline. In addition, a Shapiro-Wilk Test revealed no deviation from normality (*p* = 0.09). Week-by-week nightmare frequency exhibited some outliers and thus, nightmare frequency groups were generated to explore week-by-week changes between frequency groups rather than individuals.

Linear mixed-models (LMM) repeated measures were conducted to explore nightmare frequency and wellbeing week by week during the intervention and at the 10-month follow-up. LMM was chosen over repeated measures analysis of variance (ANOVA) because LMM can be used with data where residuals do not have constant variance across measurement points and are less affected by missing data. The repeated LMM was performed with an autoregressive covariance structure (AR1: Heterogenous), including nightmare frequency as the dependent variable, time as a fixed factor, and gender, age group, and nightmare frequency group as covariates. Significant effects were further investigated with contrast analysis using the least significant difference (LSD) adjustment. A *p*-value < 0.05 indicated statistical significance for all analyses. Separate LMMs were also conducted for Cohort 3, which included booster sessions, and Cohort 5, which included a longer-term follow-up assessment.

## Results

### Pre-treatment characteristics

#### Nightmares: frequency, duration, intensity, and characteristics by frequency groups

Descriptive statistics of the pre-treatment characteristics of nightmare frequency, duration, and intensity are presented in Table 2. Since the sample in this study was previously the subject of analyses of nightmare characteristics and academic functioning, details of some analyses are presented in that publication (Schultz et al., 2021).

**Table 2:** Descriptive statistics of the pre-treatment characteristics: Means and SD for nightmare frequency and nightmare intensity (only measured in Cohort 5).

**Characteristics**	**Females**	**Males**	**Ages 6–9**	**Ages 10–12**	**Ages 13–17**	**Total**
Frequency (nights/week)	4.84 (1.69)	4.89 (1.69)	5.00 (1.78)^**^	4.92 (1.64)	4.57 (1.69)	4.86 (1.69)
Intensity (in Cohort 5)	4.47 (1.08)	4.24 (1.11)	4.41 (0.77)	4.35 (1.18)	4.48 (1.01)	4.39 (1.10)

^**^p < 0.01.

As previously reported (Schultz et al., 2021), students in this sample reported a mean frequency of 4.86 nights with nightmares per week (SD = 1.69), with the group of 6–9-year-olds reporting a significantly higher frequency compared to the 13–17-year-olds (*p* = 0.005). With regard to nightmare duration, 26.8% of participants (292) reported 1 year or less, 21.6% (235) reported 2 years, 21.5% (234) 3 years, 11.9% (130) 4 years and 8.5% (92) 5 years and 9.6% (105) more than 5 years. Thus, 70% of participants indicated between 1 and 3 years of nightmare duration and 30% reported duration longer than 4 years. There were no differences in nightmare intensity by gender or age. In terms of other nightmare characteristics, 60.6% of the total sample had nightmares about an actual event they had experienced (that is, replay nightmares); 63.7% reported that the content reflected their “worst experience.” More than half (54.2%) had mostly the same traumatic nightmare, and 64.9% had problems going back to sleep after nightmares. As many as 56.4% reported seeing “pictures of the nightmare” during the day, indicating intrusive reexperiencing of the traumatic nightmare content. There were no significant gender or age differences in nightmare characteristics (for details, see Schultz et al., 2021). Additional results for Cohort 5, where nightmare intensity was measured by three additional items (continuous variables), showed no difference between gender or age groups (*p* = 0.14 and *p* = 0.13, respectively).

For analysis purposes, the participants were divided into three nightmare frequency groups (*N* = 1,080). The very high frequency group was defined as having nightmares six or seven nights per week and consisted of 415 participants (38.4%). The high frequency group was defined as four or five nights per week and included 488 participants (45.2%). The low/medium frequency group was defined as two or three nights per week, and 177 participants (16.4%) reported this frequency.

When comparing the nightmare frequency groups within Cohort 5, the low/medium frequency group reported higher intensity (*N* = 51, *M* = 4.56, SD = 1.00) compared to both the high frequency group (*N* = 180, *M* = 4.24, SD = 1.16) and the very high frequency group (*N* = 164, *M* = 4.49, SD = 1.04). The difference in nightmare intensity between the frequency groups was significant (*F* = 2.96, *p* < 0.05).

#### General wellbeing

Pre-treatment wellbeing was reported at a mean of 5.50 (SD = 2.82, range: 1–10). There was a significant difference between all age groups (*F* = 9.24, *p* < 0.001), with younger participants reporting higher wellbeing than older participants: 6–9-year-olds reported a mean of 6.34 (SD = 2.59), 10–12-year-olds reported a mean of 5.50 (SD = 2.84), and 13–17-year-olds reported a mean of 4.97 (SD = 2.77). There was no significant gender difference in wellbeing (*p* = 0.31). Further, the difference in wellbeing between the different nightmare frequency groups was not significant: low/medium group (*M* = 5.84, SD = 2.47), high group (*M* = 5.34, SD = 2.88), and very high group (*M* = 5.75, SD = 2.77; *F* = 2.51, *p* = 0.08).

#### Help-seeking activity

With respect to self-reported help-seeking activity, 30.3% of the participants reported having told their teacher about their nightmares pre-treatment. There were no significant differences in help-seeking activity between the nightmare frequency groups (*p* = 0.52). There was a significant difference in help-seeking activity between the age groups (*F* = 9.24, *p* < 0.001), with help-seeking decreasing with age. In 6–9-year-olds, 34.3% reported that they had spoken to their teacher about their nightmares, whereas 31.5% of the 10–12-year-old age group and 23.3% of the 13–17-year-old age group reported contacting teachers. There was no significant gender difference in help-seeking activity (χ^2^ = 10.59, df = 9, *p* = 0.30).

### Changes with treatment

#### Week-to-week changes during the intervention

**Figure 1:**
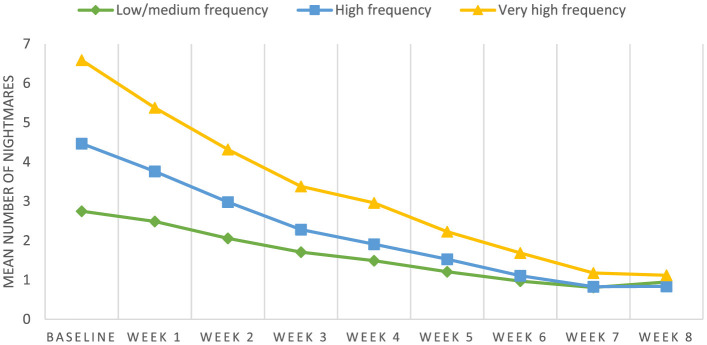
Week-by-week nightmare frequency for the three frequency groups during the intervention (*N* = 1,080). The three frequency groups were: low/medium (two or three nights per week), high frequency (four or five nights per week) and very high frequency (six or seven nights per week).

The LLM revealed an effect of time on nightmare frequency (*F* = 666.10, *p* < 0.001), with a significantly lower mean number of nightmares reported every week (*p* < 0.01) from baseline (*M* = 5.00, 1.48) to post-treatment (*M* =0.91, SD = 1.41; Cohen's *d* = 2.83, CI: 2.56–3.13, *p* < 0.001), except between the last 2 weeks of the intervention, when the number of nightmares was not significantly different (*p* = 0.43). Gender, age group, and nightmare frequency group were not found to be significant covariates (*p* = 0.94, *p* = 0.15, and *p* = 0.34, respectively). Further, there was no difference in the weekly mean number of nightmares between the different cohorts in this study (*p* = 0.67). The change in nightmare frequency over time for the three frequency groups is illustrated in Figure 1.

#### Full, partial, and non-responders to the intervention

**Figure 2:**
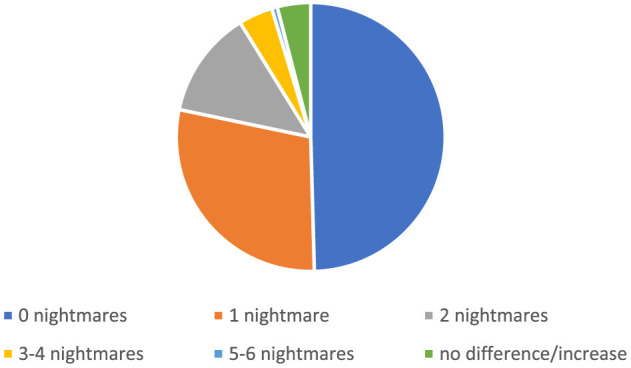
Post-treatment weekly nightmare frequency for full, partial, and non-responders.

Due to some missing data, the results of partial and non-responders to the intervention were calculated for *N* = 1,058. A total of 518 (47.96%) of the participants reported no nightmares post-treatment and were defined as full responders. Further, 498 (46.11%) reported reduced nights of nightmares after treatment and were defined as partial responders. As can be seen in Figure 2, in the partial response group, 300 (27.78%) reported one nightmare at the post-treatment assessment point, 135 (12.5%) reported two nightmares, 43 (3.98%) reported three or four nightmares, and 7 (0.65%) reported five or six nightmares in the last week. The partial responders who reported three to six nightmares post-treatment had a reduction of one to four nightmares compared to pre-test. With regard to non-responders, 19 (1.76%) of the participants reported no difference in nightmare frequency after the intervention, and 23 (2.13%) reported an increased number of nightmares at post-treatment.

There was gender balance in all response groups (full, partial, and non-responders), no statistical difference between the age groups (*p* = 0.40), and no gender difference in nightmare intensity (*p* = 0.47). Full, partial, and non-response were found in all frequency groups, as can be seen in Table 3. Finally, there was a significant difference in treatment outcomes between the nightmare frequency groups (*F* = 19.45, *p* < 0.001). Participants from the low/medium frequency group demonstrated no treatment response or increased nightmare frequency more often than those in the high frequency (*p* < 0.001) and the very high frequency groups (*p* < 0.001), suggesting a potential floor effect.

**Table 3:** Overview of the intervention response by nightmare frequency groups.

**Intervention response**	**Low/medium frequency group *N* = 175**	**High frequency group *N* = 476**	**Very high frequency group *N* = 407**	**Total *N* = 1,048**
Full response	*N* = 86 (49.1%)	*N* = 247^*^ (51.9%)	*N* = 185 (45.4%)	*N* = 518 (49.0%)
Partial response	*N* = 64 (36.6%)	*N* = 215 (45.2%)	*N* = 219^*^ (53.9%)	*N* = 498 (47.1%)
No response	*N* = 11^*^ (6.3%)	*N* = 7 (1.5%)	*N* = 1 (0.2%)	*N* = 19 (1.8%)
Increased frequency	*N* = 14^*^ (8.0%)	*N* = 7 (1.5%)	*N* = 2 (0.5%)	*N* = 23 (2.2%)

^*^p < 0.001.

#### Changes in wellbeing at 10-month follow-up (Cohort 5)

A separate LMM was performed on Cohort 5 (*n* = 215 out of 395) to explore the effect of time during the intervention and 10 months after the intervention (*F* = 115.09, *p* < 0.001). An LLM that explored changes in wellbeing in Cohort 5 revealed an effect of time (*F* = 49.61, *p* < 0.001) with significantly higher reported wellbeing 10 months post-treatment (*M* = 7.71, SD = 2.48) compared to baseline (*M* = 5.45, SD = 2.78). Gender, age group, and nightmare frequency group were not found to be significant covariates in the model (*p* = 0.50, *p* = 0.13, *p* = 0.17, respectively).

As in the LMM that included all cohorts, a significantly lower mean number of nightmares was reported every week (*p* < 0.01). The assessment at the 10-month follow-up showed no significant difference in nightmares in all frequency groups as compared to the post-treatment assessment point (*p* = 0.20), suggesting retention of the treatment gain. Only 11 participants (2.7%) were reported to need and receive one or two extra individual sessions between post-treatment and the 10-month follow-up.

**Figure 3:**
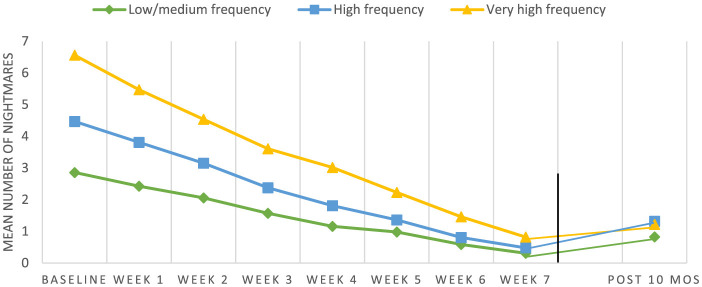
Nightmare frequency week-by-week (*N* = 395) and 10-month follow-up measure (*N* = 215) in Cohort 5. The three frequency groups were: low/medium (two or three nights per week), high frequency (four or five nights per week) and very high frequency (six or seven nights per week).

As opposed to the LMM that included all cohorts, both gender (*F* = 21.62, *p* < 0.001) and age group (*F* = 10.82, *p* < 0.001) were found to be significant covariates in Cohort 5. Males reported significantly greater reductions in nightmare frequency during the intervention (*p* < 0.01). The 6–9-year-old age group benefited more than the other age groups (*p* < 0.001). Week-by-week and post-treatment changes in nightmare frequency for the three frequency groups in Cohort 5 are illustrated in Figure 3. There were no significant differences between the frequency groups in changes in nightmare frequency.

Analyzing nightmare frequency at the follow-up in terms of responder status, of the participants who completed the 10-month follow-up assessment (*N* = 215), 70 (32.56%) reported no nightmares and 57 (26.51%) experienced one or two weekly nightmares, while 24 (11.16%) reported three to five weekly nightmares. It was further explored whether the full post-treatment responders in Cohort 5 maintained their treatment gains throughout the follow-up period. Out of the 215 participants, 137 (63.72%) had reported a full response to the intervention at post-treatment. At the 10-month follow-up, 57 (41.61%) of the 137 full responders continued to report no nightmares. Further, 57 (41.61%) reported one or two nights with nightmares per week, and 24 (17.52%) experienced three to five nightmares per week. Finally, 13 of the partial responders from this cohort reported no traumatic nightmares at the 10-month follow-up and can be characterized as late full responders.

#### Changes after post-conflict booster sessions (Cohort 3)

**Figure 4:**
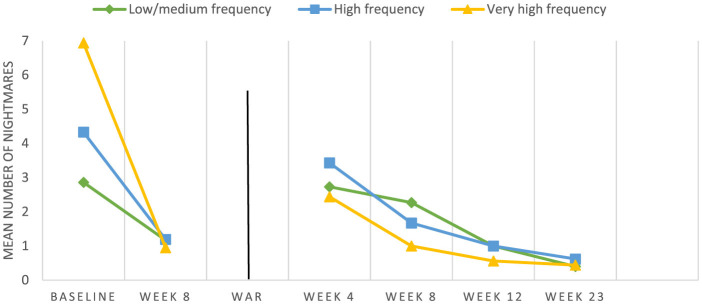
Week-by-week changes in nightmare frequency for Cohort 3 (*N* = 111), including during post-conflict booster sessions (*N* = 46). The vertical line indicates the 2014 outbreak of the 50-day armed conflict 7 weeks post-treatment. Post-conflict booster sessions were conducted at 4, 8, 12, and 23 weeks after the end of the war.

A separate LMM was performed on Cohort 3 to explore the effect of time during the intervention (*N* = 111) and the post-conflict booster sessions (*N* = 46; *F* = 62.44, *p* < 0.001). As in the larger analysis, this model found a significantly lower mean number of nightmares reported every week during the intervention from baseline (*M* = 4.61, SD = 1.69) to Week 8 (*M* = 1.10, SD = 1.09; *p* < 0.01). After Week 8, there was a significant increase in nightmares prior to the first post-conflict booster session (*M* = 3.02, SD = 1.82; *p* < 0.001). During the course of the four booster sessions, the number of nightmares was reduced session by session to below 1 per week (*M* = 0.50, SD = 0.75; *p* < 0.001). Gender, age, and nightmare frequency groups were not equally represented in the booster session sample; thus, they were not explored as covariates in the LMM. Changes in nightmare frequency over time for the three frequency groups in Cohort 3 are illustrated in Figure 4.

## Discussion

This study investigated the treatment outcome of the BLP-3, an eight-session school-based intervention for children and adolescents. A large sample of 1,093 war- and conflict-exposed students (6–17 years of age) sought help for persistent nightmares and sleep disturbance. Students reported substantial pre-treatment distress with recurrent traumatic nightmares on average four to five nights per week, with most children reporting having experienced nightmares for 1–3 years. Participants reported a significant week-to-week reduction in nightmares to an average of less than one nightmare per week after treatment, with almost half of the children experiencing no nightmares post-treatment (i.e., full responders). Improvement in nightmares was consistent from session to session, and gains were not limited to any one session; thus, students kept improving steadily. There were no significant covariates for the treatment effects; however, while only 4% failed to benefit from treatment, those with less frequent nightmares (2–3 nights per week) were more likely to be in the non-responder group after treatment. This effect may be due to the lower baseline severity and a floor effect, making it harder to detect improvement, or due to higher treatment efficacy for more severe nightmare disturbance. In terms of maintaining treatment gains, a 10-month follow-up of a smaller sample showed that 42% of full responders maintained their treatment gains and remained free from nightmares. In addition, youth reported significantly higher overall wellbeing after treatment. These findings are of particular interest considering the study was conducted in an area of ongoing armed conflict where young people have experienced very high levels of exposure to potentially traumatic events and extremely stressful conditions (e.g., United Nations Office for the Coordination of Humanitarian Affairs (UNOCHA), 2018, 2020). In addition to the major wars/scaled-up conflicts, there have been frequent armed escalations as well as ongoing life stressors related to constrained movement and inadequate resources. For example, between the end of the military operation in January 2009 and November 2012, the United Nations Office for the Coordination of Humanitarian Affairs (United Nations Office for the Coordination of Humanitarian Affairs (UNOCHA), 2013) recorded an average of 10 limited armed escalations per year in Gaza, each lasting on average 2.5 days. With regard to resources, food insecurity has been highly prevalent (Ahmadi and Melgar-Quiñonez, 2019).

In adults, imagery rescripting is the most studied and research-supported intervention for recurrent nightmares. In randomized controlled trials of imagery rehearsal for posttraumatic nightmares, effect sizes tend to be in the medium to large range (for a recent review, see Gill et al., 2023). The current study in youth showed larger effects than the adult literature has documented. Prior research of targeted treatments for nightmares in children and youth has been limited; however, several case series have suggested promising results in reducing nightmare frequency (Simard and Nielsen, 2009; St-Onge et al., 2009). The first randomized trial of a telehealth cognitive-behavioral nightmare treatment for children (CBT-NC; Cromer et al., 2024) demonstrated large reductions in nightmare frequency in children with weekly nightmares, predominantly (77%) without trauma histories. The results of Cromer et al.'s (2024) trial align with our findings of potentially larger treatment effects in a youth population. Differences in treatment delivery (the group format may have allowed for more normalization and social support), treatment content (psychoeducation and coping skills training), and treatment modality (drawing vs. writing) may all have differential, positive effects in children.

In an unplanned naturalistic experiment, one cohort in 2014 completed treatment shortly before a major 50-day armed conflict erupted that left over 2,000 Gazans dead and over 10,000 injured. We found a significant relapse in nightmare frequency from an average of 1.09 weekly nightmares after treatment to 3.2 weekly nightmares after the war. However, students with relapse responded effectively to booster sessions offered shortly after the 50-day war ended. The increase in nightmare frequency in response to additional stress and trauma was expected and is consistent with prior research suggesting that successful nightmare treatment does not “inoculate” children against future nightmares in response to additional stressors (Simard and Nielsen, 2009). The nightmare recurrence found in our study is also consistent with emotional reactions documented in research during and after the 2014 war. For example, a previous study showed that 99.1% of the students studied (*N* = 572) had experienced at least one war-related traumatic event during the 2014 war (El-Khodary and Samara, 2020). Examples of exposure included witnessing or hearing shelling by tanks, artillery, or military planes (89.3%); witnessing neighbors' houses being destroyed (69.2%); and witnessing injury or killing (66.4%). One month after the war, 57.5% of these students met the PTSD criteria. Further, students reported moderate to severe somatic symptoms (45.8%), cognitive symptoms (75.5%), emotional symptoms (72.1%), and academic dysfunction (52%) (El-Khodary and Samara, 2020). Our positive findings related to the efficacy of the booster sessions in decreasing nightmare frequency after these new potentially traumatic events are based on a small sample (*n* = 46). However, they provide a promising indication that the previously learned therapeutic strategies could be used and “reactivated” following a potential re-traumatization when prompted in a small number of group-based booster sessions. This group of students had already successfully completed their eight-session treatment, reducing nightmares from a baseline of 4.61 to 1.09 post-treatment. They had received individual tailoring of effective methods for their private toolbox, providing them with a sense of self-efficacy, control, and mastery. After being exposed to and overwhelmed by a 50-day war, these students benefited considerably from motivational support to apply previously learned methods. A systematic review of effective mechanisms of nightmare treatments (Rousseau and Belleville, 2018) supports this rationale by identifying an increased sense of mastery as one of the main mechanisms of action for reducing nightmare frequency and intensity. The current study indicates that booster sessions might be highly beneficial for maximizing the efficacy of short-term interventions in ongoing conflict settings. At the end of the 5-year implementation described in this paper, UNRWA decided to adopt the program as a standard part of the service delivered by their 150 school counselors serving 170 schools in Gaza. UNWRA described the reason for making this program available to more students as twofold: first, the high prevalence of traumatic nightmares among students, and second, the normalization of help-seeking due to the child-friendly access to school-based treatment. The program was then further contextualized and renamed to *Better Sleep—Solving Sleeping Problems for Pupils Living in Ongoing Conflict* (United Nations Relief and Work Agency for Palestinian Refugees in the Near East (UNRWA), 2017). The original manual used by NRC has recently been further revised in a new edition (Norwegian Refugee Council/Schulz Harb, 2025: 5th ed.). The strength of BLP-3 in expanding access to treatment therefore lies in both its school-based access and its focus on nightmare/sleep problems rather than on emotional/mental health symptoms that may carry greater stigma.

### Strengths and limitations of this study

The study design provides unique data on nightmares among 1,093 treatment-seeking students in Gaza. The large sample offers a broad picture that highlights prominent tendencies in treatment outcomes across age and gender and among cohorts over a 5-year period. However, we acknowledge this study's limitations. First, reliance on retrospective self-reporting may have affected measures of nightmare frequency, intensity, and duration. Even though nightmares were discussed individually when filling out the pre-treatment questionnaire, establishing nightmare duration is particularly challenging when nightmare experiences persist over several years. The measure of nightmare duration should therefore be used with caution. Secondly, the treatment outcome measure was collected by the counselor or the teacher leading the group and may have been influenced by social desirability. A stronger separation between clinical and evaluative roles or the use of blinded independent assessors would have improved credibility. Thirdly, students' PTSD symptoms were not formally assessed beyond identifying exposure to traumatic events and identifying a recurrent trauma-related nightmare. If more extensive assessment had been an option in this setting, a structured examination of both the index trauma and PTSD symptoms would have provided an interesting addition to this research. Finally, under the challenging circumstances in Gaza, a randomized controlled trial was not feasible; therefore, the Cohen's *d* within-group effect size calculated for the week-to-week changes during the intervention should be considered with caution. The unplanned naturalistic experiment with Cohort 3 pointed to an increase in nightmares during the war, followed by a decrease that appeared temporarily connected to booster sessions. Overall, however, the open-trial design of the current study, which lacked randomization and a control condition, prevents distinguishing treatment effects from natural recovery, regression to the mean, or non-specific influences (e.g., therapeutic attention or group cohesion). Combined with limited psychometric validation, and potential self-report bias, this restricts the causal interpretation of the findings.

### Further research

Several research topics should be explored in the future to further improve this program. First, it would be helpful to examine which added, supplemental, or tailored treatment is needed for students who only achieved a partial treatment gain. Second, the strong potential that the booster sessions seem to have suggests the need to test their effect and practicality rigorously going forward, not only related to new stressors but also as a standard offering within the program. Furthermore, to advance knowledge of children's functioning and development in war and conflict settings, it appears important to further investigate the hypothesis of a flow-on effect from nightmare reduction (in intensity and/or frequency) to other aspects of children's mental health. Finally, the program should be tested in a range of conflict and post-conflict settings to establish both treatment efficacy as well as feasibility and scalability, where possible in randomized controlled trials or other designs that can provide robust insights to isolate treatment effects from ancillary factors. To strengthen construct validity, future research should use standardized and validated tools (e.g., for nightmare intensity, distress and PTSD symptomatology) whenever feasible. To better understand how BLP-3 works, studies should explore mediating mechanisms such as emotion regulation, self-efficacy, or sleep quality restoration as well as analysis of moderating variables (e.g., trauma type, baseline distress, or socio-economic factors).

## Conclusions

Treatment-seeking students in this study in Gaza between 2012 and 2017 reported substantial pre-treatment distress and frequent and long-standing recurrent traumatic nightmares. The eight-session, school-based BLP-3 intervention showed promising results for reducing war- and conflict-related nightmares and increasing overall wellbeing. Half of the participating youth reported the remission of nightmares post-treatment, while an additional 47% reported a meaningful reduction in nightmare frequency. In a 10-month follow-up, treatment gains were maintained, with 42% of a smaller sample free of nightmares and 42% reporting one or two nightmares per week. Further, students with relapse after being exposed to a 50-day armed conflict responded effectively to a short series of group-based booster sessions. This indicates that war-affected children living in ongoing conflict can have significant improvement after relapse by participating in group-based booster sessions building upon previously learned therapeutic strategies. Overall, the school-based nightmare treatment demonstrates promising treatment gains for war- and conflict-affected children and youth, and the short-term program design shows promising scalability in a school environment.

## Data Availability

The raw data supporting the conclusions of this article will be made available by the authors, without undue reservation.

## References

[B1] Ahmadi D. Melgar-Quiñonez H. (2019). Determinants of food insecurity in occupied Palestinian territory: a cross-sectional survey. Lancet 393:S4. doi: 10.1016/S0140-6736(19)30590-2

[B2] Augedal A. Hansen K. Kronhaug C. Harvey A. Pallesen S. (2013). Randomized controlled trials of psychological and pharmacological treatments for nightmares: a meta-analysis. Sleep Med. Rev. 17, 143–152. doi: 10.1016/j.smrv.2012.06.001 23046846

[B3] Bernert R. A. Joiner T. E. Cukrowicz K. C. Schmidt N. B. Krakow B. (2005). Suicidality and sleep disturbances. Sleep 28, 1135–1141. doi: 10.1093/sleep/28.9.1135 16268383

[B4] Bruno W. Kitamura A. Najjar S. Seita A. Al-Delaimy W. K. (2019). Assessment of mental health and psycho-social support pilot program's effect on intended stigmatizing behavior at the Saftawi Health Center, Gaza: a cross-sectional study. J. Ment. Health 28, 436–442. doi: 10.1080/09638237.2019.1608936 31107119

[B5] Bryant B. J. (2024). Trauma exposure in migrant children: impact on sleep and acute treatment interventions. Child Adolesc. Psychiatr. Clin. N. Am. 33:193. doi: 10.1016/j.chc.2023.08.001 38395505

[B6] Casement M. D. Swanson L. M. (2012). A meta-analysis of imagery rehearsal for post-trauma nightmares: effects on nightmare frequency, sleep quality, and posttraumatic stress. Clin. Psychol. Rev. 32, 566–574. doi: 10.1016/j.cpr.2012.06.002 22819998 PMC4120639

[B7] Cohen J. A. Mannarino A. P. Deblinger E. eds. (2012). Trauma-focused CBT for Children and Adolescents: Treatment Applications. New York, NY: The Guilford Press.

[B8] Cromer L. D. Bell S. B. Prince L. E. Hollman N. El Sabbagh E. Buck T. R. (2024). Efficacy of a telehealth cognitive behavioral therapy for improving sleep and nightmares in children aged 6–17. *Front*. Sleep 3:1401023. doi: 10.3389/frsle.2024.1401023 PMC12713962 41424486

[B9] DeMarni Cromer L. Pangelinan B. A. F. Buck T. R. (2022). Case study of cognitive behavioral therapy for nightmares in children with and without trauma history. Clin. Case Stud. 21, 377–395. doi: 10.1177/15346501221081122

[B10] Dimitry L. (2011). A systematic review on the mental health of children and adolescents in areas of armed conflict in the Middle East. Child Care Health Dev. 38, 153–161. doi: 10.1111/j.1365-2214.2011.01246.x 21615769

[B11] El Sabbagh E. Johns A. N. Mather C. E. Cromer L. D. (2023). A systematic review of nightmare prevalence in children. Sleep Med. Rev. 71, 1–12. doi: 10.1016/j.smrv.2023.101834 37651893

[B12] El-Khodary B. Samara M. (2020). Effectiveness of a school-based intervention on the students' mental health after exposure to war-related trauma. Front. Psychiatry 1031, 1–10. doi: 10.3389/fpsyt.2019.01031 32273852 PMC7113368

[B13] El-Solh A. A. (2018). Management of nightmares in patients with posttraumatic stress disorder: current perspectives, Nat. Sci. Sleep 10, 409–420. doi: 10.2147/NSS.S166089 30538593 PMC6263296

[B14] Fernandez S. DeMarni Cromer L. Borntrager C. Swopes R. Hanson R. F. Davis J. L. (2013). A case series: cognitive-behavioral treatment (exposure, relaxation, and rescripting therapy) of trauma-related nightmares experienced by children. Clin. Case Stud. 12, 39–59. doi: 10.1177/1534650112462623

[B15] Gill P. Fraser E. Tran T. T. D. De Sena Collier G. Jago A. Losinno J. . (2023). Psychosocial treatments for nightmares in adults and children: a systematic review. BMC Psychiatry 23:283. doi: 10.1186/s12888-023-04703-1 37085821 PMC10122409

[B16] Harb G. C. Cook J. M. Gehrman P. R. Gamble G. M. Ross R. J. (2009). PTSD nightmares and sleep disturbance in Iraq war veterans: a feasible and promising treatment combination. J. Aggress. Maltreat. Trauma 18, 516–531. doi: 10.1080/10926770903035150

[B17] Harb G. C. Schultz J. H. (2020). Posttraumatic nightmares and school functioning in war-affected youth. PLoS ONE 15:e0242414. doi: 10.1371/journal.pone.0242414 33237929 PMC7688112

[B18] Hobfoll S. E. Watson P. Bell C. C. Bryant R. A. Brymer M. J. Friedman M. J. (2007). Five essential elements of immediate and mid-term mass trauma intervention: empirical evidence. Psychiatry 70, 283–315. doi: 10.1521/psyc.2007.70.4.283 18181708

[B19] Kadir A. Shenoda S. Goldhagen J. (2019). Effects of armed conflict on child health and development: a systematic review. PLoS ONE 14:e0210071. doi: 10.1371/journal.pone.0210071 30650095 PMC6334973

[B20] Kovachy B. O'Hara R. Hawkins N. Gershon A. Primeau M. M. Madej J. . (2013). Sleep disturbance in pediatric PTSD: current findings and future directions. J. Clin. Sleep Med. 9, 501–510. doi: 10.5664/jcsm.2678 23674943 PMC3629326

[B21] Krakow B. Sandoval D. Schrader R. Keuhne B. McBride L. Yau C. L. . (2001). Treatment of chronic nightmares in adjudicated adolescent girls in a residential facility. J. Adolesc. Health 29, 94–100. doi: 10.1016/S1054-139X(00)00195-6 11472867

[B22] Levin R. Nielsen T. A. (2007). Disturbed dreaming, posttraumatic stress disorder, and affect distress: a review and neurocognitive model. Psychol. Bull. 133, 482–528. doi: 10.1037/0033-2909.133.3.482 17469988

[B23] Nader K. (1996). “Children's traumatic dreams,” in Trauma and Dreams, ed. D. Barrett (Harvard University Press), 9–24.

[B24] Norwegian Refugee Council (2011). Better Learning Programme 3: Better Sleep by Facing Your Nightmares—Group and Individual Sessions, 2nd edn. Oslo, Norway: UiT, the Arctic University of Norway & Norwegian Refugee Council.

[B25] Norwegian Refugee Council (2022). Better Learning Program 2: Group Sessions. Improving Study Skills, Education in Emergencies, 3rd edn. Oslo, Norway: Department of Education, UiT, the Arctic University of Norway & Norwegian Refugee Council.

[B26] Norwegian Refugee Council (2025). Better Learning Programme 1: Supporting Students' Recovery in Emergencies, Classroom Sessions, 5th edn. Oslo, Norway: Department of Education, UiT, the Arctic University of Norway & Norwegian Refugee Council.

[B27] Norwegian Refugee Council/Schulz and Harb (2025). Better Learning Programme 3: Better Sleep by Facing Your Nightmares—Group and individual Sessions, 5th edn. Oslo, Norway: UiT Arctic University of Norway & Norwegian Refugee Council.

[B28] Ossa F. C. Bering R. Pietrowsky R. (2013). Prevalence and intensity of nightmares in traumatized and nontraumatized children and adolescents. Z. Kinder Jugendpsychiatr. Psychother. 41, 309–317. doi: 10.1024/1422-4917/a000246 23988833

[B29] Pruiksma K. E. Miller K. E. Davis J. L. Gehrman P. Harb G. Ross R. J. . (2025). An expert consensus statement for implementing cognitive behavioral therapy for nightmares in adults. Behav. Sleep Med. 23, 1–9. doi: 10.1080/15402002.2024.2437634 39815631

[B30] Ross R. J. Ball W. A. Sullivan K. A. Caroff S. N. (1989). Sleep disturbance as the hallmark of posttraumatic stress disorder. Am. J. Psychiatry 146, 697–707. doi: 10.1176/ajp.146.6.697 2658624

[B31] Rousseau A. Belleville G. (2018). The mechanisms of action underlying the efficacy of psychological nightmare treatments: a systematic review and thematic analysis of discussed hypotheses. Sleep Med. Rev. 39, 122–133. doi: 10.1016/j.smrv.2017.08.004 29056416

[B32] Schauer M. Neuner F. Elbert T. (2005). Narrative Exposure Therapy: A Short-Term Intervention for Traumatic Stress Disorders after War, Terror, or Torture. Cambridge, MA: Hogrefe & Huber.

[B33] Schredl M. Reinhard I. (2011). Gender differences in nightmare frequency: a meta-analysis. Sleep Med. Rev. 15, 115–121. doi: 10.1016/j.smrv.2010.06.002 20817509

[B34] Schultz J.-H. Forsberg J. Harb G. Alisic E. (2021). Prevalence and characteristics of post-traumatic nightmares in war- and conflict-affected students. Nat. Sci. Sleep 13, 423–433. doi: 10.2147/NSS.S282967 33776500 PMC7989377

[B35] Secrist M. E. John S. G. Harper S. L. Conners Edge N. A. Sigel B. A. Sievers C. . (2019). Nightmares in treatment-seeking youth: the role of cumulative trauma exposure. J. Child Adolesc. Trauma 13, 249–256. doi: 10.1007/s40653-019-00268-y 32549936 PMC7289908

[B36] Simard V. Nielsen T. (2009). Adaptation of imagery rehearsal therapy for nightmares in children: a brief report. Psychotherapy 46, 492–497. doi: 10.1037/a0017945 22121846

[B37] St-Onge M. Mercier P. De Koninck J. (2009). Imagery rehearsal therapy for frequent nightmares in children. Behav. Sleep Med. 7, 81–98. doi: 10.1080/15402000902762360 19330581

[B38] United Nations Human Rights Council (UNHRC) (2015). Report of the Independent Commission of Inquiry on the 2014 Gaza Conflict. A/HRC/29/52. Available online at: https://www.ohchr.org/en/hrbodies/hrc/coigazaconflict/pages/reportcoigaza.aspx (Accessed May 15, 2025).

[B39] United Nations Human Rights Council (UNHRC) (2022). United Nations Human Rights Report 2022. Geneva, Switzerland: UN Human Rights Council, Office of the High Commissioner.

[B40] United Nations Office for the Coordination of Humanitarian Affairs (UNOCHA) (2013). Fragmented Lives: Humanitarian Overview. Jerusalem, Israel: UNOCHA. Available online at: https://www.ochaopt.org/sites/default/files/ocha_opt_fragmented_lives_annual_report_2013_english_web.pdf (Accessed May 15, 2025).

[B41] United Nations Office for the Coordination of Humanitarian Affairs (UNOCHA) (2018). Humanitarian Needs Overview. Occupied Palestinian Territory. Jerusalem, Israel: UNOCHA.

[B42] United Nations Office for the Coordination of Humanitarian Affairs (UNOCHA) (2020). Overview: 2010–2019: A Decade in Numbers. Jerusalem, Israel: UNOCHA. Available online at: https://www.ochaopt.org/page/publications1

[B43] United Nations Relief and Work Agency for Palestinian Refugees in the Near East (UNRWA) (2017). Better Sleep—Solving Sleeping Problems for Pupils Living in Ongoing Conflict. Gaza, Palestine: UNRWA.

[B44] Wamser-Nanney R. Chesher R. E. (2017). Trauma characteristics and sleep impairment among trauma-exposed children. Child Abuse Negl. 76, 469–479. doi: 10.1016/j.chiabu.2017.11.020 29268207

