# Supplementary material for: Treating war- and conflict-related nightmares in children and youth: outcomes of a school-based intervention
Source: Front Sleep. 2026 Jan 8;4:1658555. doi: 10.3389/frsle.2025.1658555 (PMC12823506; doi:10.3389/frsle.2025.1658555)
Supplement: Supplementary file 1 [file Table_1.docx]

BLP-3: Better Sleep by Facing Your Nightmares

Group and Individual Sessions

The program involves 8 sessions: 4 group sessions, 4 individual sessions. The core program should preferably be completed within a period of 8 weeks. Each of the eight sessions is manualized with a detailed structure. Below is a general overview.

**Part 1: Four Group Sessions**

The program involves four group sessions (5-10 children). Their objective is to understand the nature of traumatic events, understand and recognize reactions to traumatic stress, and learn strategies to reduce the intensity and frequency of nightmares.

General structure of all group sessions:

- Introduction to session.
  - Motivation building & relaxation practice.
- Teaching of new information/skill.
- Checking in.
- Closing.
  - Reinforcing learners’ efforts, looking ahead to next session.
  - Assigning homework, sing theme song.

Topics:

- Information about traumatic stress reactions and what causes such symptoms.
- Relaxation techniques that may calm the body and mind.
- Normalizing of nightmare experiences: Frequency and content.

**Part 2: Four Individual Sessions**

The program also involves four individual sessions. Their objective is to further reinforce and adapt personal routines for relaxation exercises. We draw a timeline of the events in learners’ lives, and we identify the most troublesome nightmare, we draw it and change it by making a new drawing.

When people are exposed to traumatic events, they tend to lose sight of their timeline and the sequence of events in their lives becomes blurry. By reconstructing the learners’ timeline (including good events and bad) and by drawing the bothersome nightmare, we connect the fragmented memories to words in order to make the traumatic experiences understandable events belonging to the past. Finally, by changing the nightmare story into a less distressing dream story, learners will experience more control over their dream and rehearse a new, more positive, dream story before bed.

Topics:

- Individual timeline of life events.
- Retelling/drawing of target nightmare.
- Rescripting/drawing new nightmare story.
- Reinforcing individual sleep routines.
